# Targeted exome sequencing of unselected heavy‐ion beam‐irradiated populations reveals less‐biased mutation characteristics in the rice genome

**DOI:** 10.1111/tpj.14213

**Published:** 2019-02-25

**Authors:** Hiroyuki Ichida, Ryouhei Morita, Yuki Shirakawa, Yoriko Hayashi, Tomoko Abe

**Affiliations:** ^1^ RIKEN Nishina Center for Accelerator‐Based Science Wako Saitama 351‐0198 Japan

**Keywords:** heavy‐ion beam, mutagenesis, whole‐exome sequencing, rice (*Oryza sativa*), mutation rate

## Abstract

Heavy‐ion beams have been widely utilized as a novel and effective mutagen for mutation breeding in diverse plant species, but the induced mutation spectrum is not fully understood at the genome scale. We describe the development of a multiplexed and cost‐efficient whole‐exome sequencing procedure in rice, and its application to characterize an unselected population of heavy‐ion beam‐induced mutations. The bioinformatics pipeline identified single‐nucleotide mutations as well as small and large (>63 kb) insertions and deletions, and showed good agreement with the results obtained with conventional polymerase chain reaction (PCR) and sequencing analyses. We applied the procedure to analyze the mutation spectrum induced by heavy‐ion beams at the population level. In total, 165 individual M_2_ lines derived from six irradiation conditions as well as eight pools from non‐irradiated ‘Nipponbare’ controls were sequenced using the newly established target exome sequencing procedure. The characteristics and distribution of carbon‐ion beam‐induced mutations were analyzed in the absence of bias introduced by visual mutant selections. The average (±SE) number of mutations within the target exon regions was 9.06 ± 0.37 induced by 150 Gy irradiation of dry seeds. The mutation frequency changed in parallel to the irradiation dose when dry seeds were irradiated. The total number of mutations detected by sequencing unselected M_2_ lines was correlated with the conventional mutation frequency determined by the occurrence of morphological mutants. Therefore, mutation frequency may be a good indicator for sequencing‐based determination of the optimal irradiation condition for induction of mutations.

## Significance Statement

We developed a multiplexed and cost‐efficient whole‐exome sequencing procedure and bioinformatics filtering pipeline for rice, and demonstrated the rapid identification of authentic mutations from a large dataset of candidate mutations. We applied this technique for the characterization of mutation spectra by heavy‐ion beams in the absence of the mutant selection, and showed that 9.06 ± 0.37 (average ± SE) mutations were induced within the target exon regions by 150 Gy irradiation of dry seeds in rice.

## Introduction

Mutagenesis is a fundamental tool with which to investigate gene functions and create new cultivars in plant breeding. Mutations can be induced artificially by chemical mutagens or arise naturally as the result of cellular activity such as radicals produced in respiration and errors in DNA duplications as well as natural radiation. Among the mutagens that have been used to induce mutations, chemical mutagens such as ethyl methane sulfonate (EMS), or ionizing radiation such as X‐rays or γ‐rays, have been extensively utilized in the mutagenesis of diverse plant species (Mutant Variety Database; https://mvd.iaea.org/). A heavy‐ion beam is a type of ionizing radiation that is produced by a heavy‐ion accelerator, such as a cyclotron or synchrotron, and is known to cause DNA double‐stranded breaks in a cell in the proximity of the beam tracks. Mutations such as insertions, deletions, inversions, and base substitutions in the genome. The radioactive isotope beam factory (RIBF) at the RIKEN Nishina Center for Accelerator‐Based Science is one of the largest and most powerful heavy‐ion accelerator facilities in the world. Accelerated heavy‐ion beams have sufficient energy to penetrate plant cells and tissues in air; for example, the range for carbon ions (^12^C^6+^, 135 MeV nucleon^−1^) extends to 40 mm in water (Ryuto *et al*., 2008). It is well known that a carbon‐ion beam is a suitable mutagen for use in plant breeding, because new cultivars with desired traits can be generated by irradiation at a relatively low dose, which does not affect other traits (Tanaka *et al*., 2010; Abe *et al*., 2012, 2015).

We have adopted several approaches to study the molecular nature of heavy‐ion beam‐induced mutations, including single‐gene analysis by PCR and sequencing (Kazama *et al*., 2011; Hirano *et al*., 2012) and genome‐wide analysis of large deletions by array‐comparative genomic hybridization (Kazama *et al*., 2013; Hirano *et al*., 2015) in the dicotyledonous model plant Arabidopsis (*Arabidopsis thaliana*). Carbon‐ion beam irradiation shows a high mutation efficiency (Kazama *et al*., 2008), and mainly induces base substitutions and deletions and insertions of less than 100 bp (Kazama *et al*., 2011). Whole‐genome sequencing (WGS) is a useful tool to analyze the genome‐wide nature of the induced mutations and the mutation frequency. In Arabidopsis, the mutagenic effect of carbon‐ion beams has been investigated by WGS, which has confirmed that carbon‐ion irradiation frequently induces single‐base substitutions and small deletions and insertions in a genome‐wide manner. The average number of mutated genes, including both homozygous and heterozygous mutations, was 13 in each M_3_ mutant (Kazama *et al*., 2017; Du *et al*., 2018). In the case of randomly chosen M_2_ lines, an average of 7.0 genes were affected as homozygous mutations (Hase *et al*., 2018). In addition, relatively large deletions and genomic rearrangements, such as inversions and translocations, were also detected by WGS of selected carbon‐ion irradiation‐derived mutants. Overall, a relatively small number of mutations were detected in the M_3_ generation, which is consistent with the expectation from previous observations that highly accelerated heavy‐ion particle irradiation induces localized double‐stranded breaks of DNA (Nakajima *et al*., 2013).

Recent advances in massively parallel (or next‐generation) DNA sequencing technology enable comprehensive analysis of genomic mutations at base‐pair resolution. Rice (*Oryza sativa*) has a relatively small genome (389 million bases per haploid) among crops in general, and much of the genome consists of repetitive and junk elements and only ~10% (41.51 Mb in the Os‐Nipponbare‐Reference‐IRGSP‐1.0 [IRGSP‐1.0] genome annotation) is predicted to encode proteins. Target capture by hybridization is an effective means to concentrate the regions of interest from a randomly sheared, unbiased sequencing library. In this manner, a large set of DNA or RNA oligonucleotide probes, which are complementary to the target sequences and include modified moieties for selective capture in solution (e.g. biotinylated oligonucleotide probes and streptavidin‐coated magnetic particles), was used to concentrate the fragments with the sequences of interest from the DNA library. In whole‐exome sequencing, probes that are complementary to each of the exons encoded in the genome are synthesized, hybridized with the randomly sheared genomic DNA libraries, then the resulting exon‐enriched library is subjected to sequencing (Bamshad *et al*., 2011). One downside of this approach is the relatively high cost of target enrichment and predominantly the probes, therefore multiple libraries with different indices are pooled prior to the target capture (pre‐capture multiplexing), then subjected to hybridization to reduce the cost per sample. For a small number of targets (total target length of 521 467 bp), pre‐capture multiplexing of as many as 16 different libraries can be achieved without the loss of capture specificity (Shearer *et al*., 2012). In plants, Henry *et al*. (2014) reported large‐scale exome sequencing of EMS‐treated M_2_ plants in rice and hexaploid wheat (*Triticum aestivum*). These authors identified ~18 000 mutations from 72 individuals and estimated that deleterious mutations were induced in more than 2600 genes. These reports indicated that whole‐exome sequencing is a realistic approach to achieve comprehensive genome‐wide analysis of genomic mutations caused by heavy‐ion beams at the population scale. Previous studies of Arabidopsis have revealed molecular characteristics of heavy‐ion‐induced mutations in selected morphological mutants. However, the nature of heavy‐ion beam‐induced mutations in the absence of bias from the selection of mutants remains unknown.

In the present study, we developed a multiplexed and cost‐efficient whole‐exome sequencing procedure in rice. The designed probe set, SeqCap™ custom design number OID37934, covers more than 95% of the protein‐coding regions defined in the IRGSP‐1.0 annotation. We applied a ‘pre‐capture multiplexing’ technique and determined the spectrum of heavy‐ion beam‐induced mutations in 165 individual carbon‐ion beam‐irradiated and unselected M_2_ lines as well as eight pools of the corresponding non‐irradiated rice ‘Nipponbare’ controls. For this purpose, we also developed an efficient bioinformatics pipeline to extract authentic mutations induced by heavy‐ion beams from a large number of candidate mutations, mostly due to intracultivar variations of Nipponbare between the sequenced strain and the strain maintained in our laboratory. We report the comprehensive profiling of heavy‐ion beam‐induced mutations in a large and unbiased rice population, and provide fundamental insights to investigate the mechanisms of mutagenesis and repair of the genomic lesions caused by high‐energy heavy‐ion beams in plants.

## Results

### Rice whole‐exome enrichment using custom‐designed hybrid sequence‐capturing probes

We employed rice exon enrichment using a custom‐designed, commercially available target enrichment platform (SeqCap EZ developer library). The probes were designed based on the sequences and annotations in IRGSP Build 5 (http://rapdb.dna.affrc.go.jp/download/build5.html), which was the commonly used rice reference sequence at the time of our initial production. We aimed to enrich a total region of 41.75 Mb that spanned coding sequences and their flanking regions. We mapped the nucleotide sequences of the target regions in Build 5 to the latest IRGSP‐1.0 sequences (Kawahara *et al*., 2013) using BLAST‐N (Camacho *et al*., 2009) with an *E*‐value of less than 1.0 × e^−10^ as the threshold for significant hits. We assumed the IRGSP‐1.0 region with the highest identity is the corresponding query locus in the Build 5 sequence because the nucleotide sequences are extremely similar in most parts of the genome, particularly in coding regions. Although the BLAST‐N search successfully identified at least one candidate location for 300 085 of the 300 747 regions (99.78%), there were records indicating inter‐ and intrachromosomal translocations that were not reported in the building process from Build 5 to IRGSP‐1.0. We hypothesized that this was attributed to incorrect translation, therefore we implemented a program to fine‐tune and verify the correspondence based on the sequence identities and synteny between the Build 5 and IRGSP‐1.0 sequences. As a result, only one locus in the Build 5 sequence was not located in the IRGSP‐1.0 sequences; all other targets were successfully located in the IRGSP‐1.0 sequences (Data S1). All subsequent analyses were based on IRGSP‐1.0 sequences and the translated enrichment target locations.

We tested the efficiency of the target enrichment and aimed to identify the mutated genes in previously isolated rice mutants that were generated by heavy‐ion beam irradiation and subsequent selfing. For this purpose, seven individual mutants isolated after carbon‐ion beam irradiation, namely 3‐14 (ripening failure), 3‐51 (dwarf), 4‐13 (weak growth), 5‐12 (stripe), 6‐99 (salt tolerance), 14‐45 (salt tolerance), and 18‐36 (salt tolerance), as well as five mutants isolated following neon‐ion beam irradiation, namely 6‐62 (dwarf), 7‐30 (pale green), 7‐3B (lesion mimic), 14‐45 (salt tolerance), and Ne‐1779 (virescent), were subjected to target exon enrichment and sequencing. In this initial analysis, the sequencing library was produced from randomly sheared genomic DNA of each mutant, subjected to individual target exon enrichment (no pre‐capture multiplexing), and then an equal molar amount from each sample was pooled and sequenced with the objective to enrich more than 100 times equivalent of the target exon regions. We aimed to enrich 41 748 624 bases spanning 300 746 regions, defined based on the gene definition in Build 5. The sequencing statistics are shown in Table S1. We successfully obtained 5.64–9.98 billion bases from each mutant, and almost or more than 99% of these bases were mapped on IRGSP‐1.0 sequences. The total amount of sequence obtained in the initial analysis was equivalent to 15.10–26.74 times that of the whole rice genome but, as expected, the mean read coverage in the target regions was much higher (69.15–127.74), which indicated that the defined target region was successfully enriched in the process.

To minimize nonspecific hybridization, we prepared a rice repetitive DNA fragment library, which corresponded to human Cot‐1 DNA, a placental DNA enriched with repetitive sequences of Alu and Kpn family members of 50–300 bp in length, which is often used as a blocking agent during hybridization. When comparing two different blocking agents (‘Blocking DNA’ in Table S1), the in‐house‐prepared rice repetitive DNA fragment library and a commercial universal blocking reagent (SeqCap EZ Developer Reagent; ‘SeqCap’ in Table S1), the rice repetitive DNA provided slightly better target coverage than that of the universal blocking reagent. However, production of the rice repetitive DNA fragment library requires a long lead‐time and is labor intensive, therefore we used the commercial blocking reagent in the following experiments.

### Exome sequencing in combination with bioinformatics filtering enables rapid identification of mutated genes

In the present target exome sequencing, we aimed to obtain approximately 100 times the equivalent amount of sequencing reads (~4 billion bases as quality‐filtered reads) from each target‐enriched sample. The reads were then mapped on the IRGSP‐1.0 sequences and subjected to variant calling with the programs Genome Analysis Toolkit (GATK; McKenna *et al*., 2010) and Pindel (Ye *et al*., 2009). Initially, we simply combined the raw outputs from these programs to identify possible mutations that were induced by heavy‐ion beam irradiation and subsequent DNA repair. The combination of these programs typically listed more than 10 000 variants in each line, and most of these variants were common among independently obtained mutants (Table 1). The Nipponbare cultivar contains multiple intracultivar genotypes that may reflect residual heterozygosity, outcrossing, and somatic mutations during propagation in different locations (Kawahara *et al*., 2013). Given that all of the present mutants were induced from a single Nipponbare variant, these common variants were due to intracultivar genetic differences between the Nipponbare strain used in the present study and the genotype sequenced in the rice genome project. Therefore, we implemented a filtering program in our bioinformatics pipeline to reduce false positives caused by intracultivar variation, which is conceptually similar to a previously reported bioinformatics pipeline for Arabidopsis (Ishii *et al*., 2016). Briefly, we assumed an identical mutation that is shared among two or more mutant lines is intracultivar variation, and implemented a filtering program in the pipeline to remove those mutations from the raw variant calling results. We also removed records with more than 5% of reference‐type reads and with multiple alleles because these are often caused by mapping errors in and near repeated sequences. This filtering strategy worked extremely well and reduced the number of candidate records to less than 1% (Table 1). To test the validity of the filtering and thresholds, we used the combination of PCR and dideoxy DNA sequencing to determine if the reported variant was present in the mutant genome. We performed this test against all reported mutations, regardless of whether the mutation was located in the target regions or not, that passed the filtering in eight mutants (3‐14, 3‐51, 4‐13, 5‐12, 6‐62, 7‐30, 7‐3B, and Ne‐1779). Of the 114 mutations detected in the pipeline, we achieved specific amplification of 106 loci by PCR. Among these 106 loci, 91 mutations (85.8%) were confirmed by PCR and conventional dideoxy sequencing, whereas the remaining 15 mutations (14.2%) were not present in the genome (Table S2). The percentage of real mutations, which were detected by the pipeline and confirmed by PCR and dideoxy sequencing, was in a similar range regardless of the mutation type. In the case of single‐nucleotide variants (SNVs), 67 mutation candidates were detected by the pipeline, and 56 (83.6%) of these were confirmed to exist in the mutant genome by PCR and dideoxy sequencing. Similarly, the percentages of real mutations were 87.0% (20 out of the 23 candidates detected) and 83.3% (five out of the six candidates detected) for deletions and insertions, respectively. The sizes of the detected and confirmed mutations were 1–212 bp for deletions and 1–15 bp for insertions. In the case of inversions, replacements, and tandem duplications, all mutation candidates detected by the pipeline were present in the mutant genome, although the numbers of detected mutations were relatively small for these types of mutations. These results showed that the filtering strategy implemented in the pipeline provided reasonable accuracy and specificity in exome sequencing analysis in rice, and that the combination of whole‐exome sequencing and the bioinformatics pipeline could detect SNVs as well as relatively small (≤200 bp) deletions and insertions induced by carbon‐ion beam irradiation.

**Table 1:** Efficiency of the filtering strategy based on mutant line specificity

Line	GATK			Pindel			Number of unique mutations
	Raw output	Common variants^a^	Filtered result	Raw output	Common variants^a^	Filtered result	
Carbon‐ion induced mutations							
3‐14	1355	1330	6	10 988	10 669	3	8
3‐51	1326	1299	7	11 515	10 775	7	12
4‐13	1265	1258	3	11 048	10 681	3	6
5‐12	1190	1184	3	10 903	10 632	2	4
6‐99	1290	1256	4	2316	1918	4	8
14‐45^b^	21 959	1531	19 722	58 752	53 049	3890	22 115
18‐36	1298	1252	8	2319	1918	4	12
Neon‐ion induced mutations							
6‐62	1356	1337	8	9396	9097	4	9
7‐30	1366	1354	2	9415	9137	3	5
7‐3B	1367	1339	8	9310	9044	8	14
Ne‐1779	1124	1111	3	9407	9213	7	10

a Variants that were common between two or more of the mutants analyzed.

b Probable outcrossing or seed contamination.

**Figure 1:**
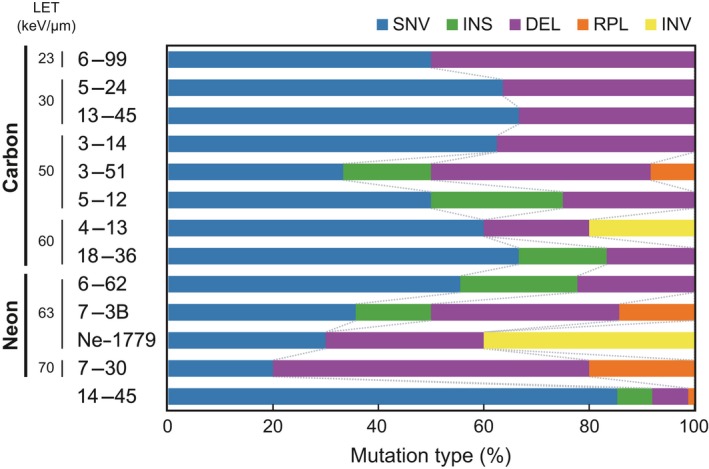
Mutation types detected in 13 heavy‐ion beam‐induced mutants of rice The proportion of each type of mutation (SNV, single‐nucleotide variants; DEL, deletions; INS, insertions; RPL, replacements; INV, inversions) is shown. Targeted exome sequences were obtained for 11 mutants, induced by carbon‐ion and neon‐ion irradiation, followed by variant calling with the Genome Analysis Toolkit and Pindel programs integrated into the bioinformatics pipeline. Note that the 14‐45 mutant arose by seed contamination or outcrossing.

The types of mutations detected using the bioinformatics pipeline are summarized in Figure 1. In the 13 mutants tested, the most frequent type of mutation in an individual mutant was SNVs (20.0–85.4%) in a majority of the mutants, regardless of their phenotypes and differences in irradiation dose and linear energy transfer (LET). In the case of mutants 6‐99, 3‐51, 7‐3B, and 7‐30, deletions followed by SNVs were the most frequent types of mutations present. Replacements and inversions were less frequent in all mutants, although this may be attributed to the fact that detection of these types of mutations from short‐read datasets is inefficient. Overall, no significant difference was observed in the composition of the induced mutations with differences in LET, the irradiated ion, or the irradiation dose at the exome level. These results indicated that the performance of target exome capturing and the bioinformatics analysis was not affected by such differences in physical parameters; therefore, our target exome sequencing and bioinformatics approach was suitable for analyzing the gene‐wide characteristics of the induced mutations under different physical and physiological conditions.

After the initial analysis, we unexpectedly established that one of the mutants tested, 14‐45, which was isolated as a salt‐tolerant mutant, was an accidental hybrid between Nipponbare and an unknown cultivar/strain, presumably grown in the vicinity of our saline paddy field. Although rice is self‐fertile, the frequency of outcrossing often increases in mutants, especially when fertility is affected (Fu *et al*., 2008). The bioinformatics pipeline detected an unusually high number of mutations from this line (34 924 mutations in total from GATK and Pindel after filtering, compared with equal or less than 14 mutations in all other mutants). Despite the high frequency of base substitutions and other structural mutations, the target exon enrichment and mutation detection had been successful, and the quality of results was comparable in terms of the read depth in the target regions (Table S1). Therefore, we concluded that the target exon enrichment and bioinformatics pipeline developed in the present study is applicable to a wide range of rice cultivars, and presumably both *japonica* and *indica* subspecies of rice.

As a trade‐off to efficiently target the mutations that are likely to affect protein functions in target‐enriched sequencing, identification of relatively large deletions is problematic, particularly for deletions spanning one or more entire exons. This is because the upstream and downstream edges of a deletion must be present within the target regions to be detected as chimeric fragments, which indicate deletions, otherwise this evidence is lost during target enrichment. Therefore, we introduced an approach to detect large deletions, spanning one or more entire target exon regions, by extracting the target regions that were not covered by the mapped reads. For this purpose, we utilized the ‘coverage’ tool in Bedtools and the translated target coordinates. Although this approach is prone to select false positives because there is no way to change the threshold read count and obtain detailed statistics, it enabled detection of a number of large deletions from the exome‐sequencing results. In particular, the analysis revealed a large (>63 kb) deletion in the 6‐62 mutant that spanned 35 consecutive exons between positions 3 063 815 and 3 127 160 on chromosome 9 (Table S3). After manual inspection and subsequent PCR amplification and sequencing analysis (Figure S1), the deletion was determined to be 102 158 bp in length and caused the loss of seven genes (Os09g0240200–1200), which are predicted to encode zinc finger protein with B‐box domain (Os09g0240200), chloroplast precursor of sulfate transporter 4.1 (Os09g0240500), nucleotide‐binding protein (Os09g0241100), and hypothetical proteins (Os09g0240350, Os09g0240850, Os09g0240975, and Os09g0241200). Therefore, we speculated that one or more of the genes within the deletion caused the dwarf phenotype. The mutants 7‐30, 7‐3B, and Ne‐1779 were revealed to have suffered deletions of 79 bp (positions 1 154 550–1 154 629 on chromosome 6), 450 bp (positions 4 215 915–4 216 365 on chromosome 12), and 40 784 bp (positions 14 871 045–14 911 829 on chromosome 10) by our coverage analysis program, respectively. Among these deletions, the 40 784 bp deletion identified for the Ne‐1779 mutant matched the results from genetic mapping, and we eventually showed that this deletion spans 46.4 kb in length. These results indicated that the genes responsible for the virescent phenotype observed in the Ne‐1779 mutant may be located within the deletion. A gene encoding a pentatricopeptide repeat domain‐containing protein, Os10g0421800, which is indicated to be an ortholog of Arabidopsis proton gradient regulation 3 (*PGR3*) that causes aberrant chlorophyll fluorescence (Yamazaki *et al*., 2004), is located within the deletion, therefore we hypothesized that this gene may be involved in the virescent phenotype of the Ne‐1779 mutant. From these results, it is evident that large deletions caused by accelerated heavy‐ions can be detected by target sequencing using a newly developed coverage calculation program.

**Figure 2:**
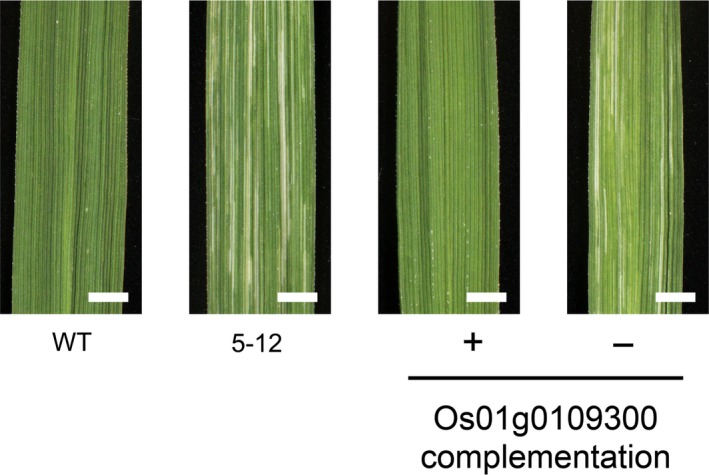
Phenotypes of the 5‐12 mutant and its transgenic derivatives Leaf blades were sampled from advanced‐stage plants and imaged under white light‐emitting diode (LED) illumination. wild‐type (WT): Nipponbare (wild‐type), 5‐12: a striated‐leaf mutant isolated from a population irradiated with carbon‐ion beams, Os01g0109300 complementation +: genomic Os01g0109300 fragment introgressed by *Agrobacterium*‐mediated transformation, Os01g0109300 complementation −: a segregate progeny of a transformed 5‐12 plant lacking the introgressed Os01g0109300 fragment. Bar: 2 mm.

This ‘genome‐landing’ approach is also useful for the cloning of unknown genes. The mutant 5‐12, which shows many white striations or stripes on the leaves at an advanced stage of growth, carried four mutations in the target exon regions. Two of these mutations were estimated to be homozygous and consisted of an SNV (C to A substitution), which causes a mis‐sense mutation in the amino acid sequence, and an 18‐bp deletion, which causes an in‐frame deletion in the coding sequence. The other two heterozygous mutations were a mis‐sense mutation (A to G substitution) and a 1‐bp (T) insertion at the putative promoter region. We tested for genetic linkage between these four mutations and the stripe phenotype using a small number of F_2_ progenies (~16 individuals) derived from a cross between the 5‐12 mutant and wild‐type Nipponbare. The genetic linkage analysis revealed that the 18‐bp deletion occurred within the coding sequence of Os01g0109300 linked to the striated‐leaf phenotype, therefore it was possible that this gene is responsible for the phenotype. The gene was annotated as ‘similar to predicted protein’ in the rice annotation project database (RAP‐DB; http://rapdb.dna.affrc.go.jp/) and showed no similarity with known proteins and domains. To further confirm that the gene Os01g0109300 is responsible for the striated‐leaf phenotype, we created a transgenic rice plant that introgressed a genomic Os01g0109300 fragment from the wild‐type Nipponbare into the 5‐12 genome. As shown in Figure 2, the leaves of the transgenic plant showed complete recovery of the striated‐leaf phenotype, whereas the segregated sibling lacking the transgene showed the striated‐leaf phenotype, which revealed that the loss of function for Os01g0109300 caused the striated‐leaf phenotype in the 5‐12 mutant. Therefore, it is evident that an approach that combines target exome sequencing and genetic linkage analysis with a small segregating population is a powerful tool to rapidly identify responsible genes that do not show any similarity with previously identified genes in rice. This approach is particularly useful when the introduced mutations are deletions and insertions: one can use the mutations as indel markers, which can easily determine the genotype by high‐resolution capillary gel electrophoresis, in the linkage analysis.

### Pre‐capture multiplexing enables population‐scale mutant analysis at reduced cost

Target enrichment reduced the sequencing cost significantly, but the cost of target enrichment probes assumed a significant portion of the total cost. Several previous reports have utilized pre‐capture multiplexing, which prepares a sequencing library individually from each sample with different index (barcode) sequences, then the prepared libraries are pooled and subjected to target enrichment as a mixture, but mostly for a relatively small number of targets (Rohland and Reich, 2012; Shearer *et al*., 2012; van der Werf *et al*., 2015). In this manner, the cost per sample for reagents, consumables, and labor in the target enrichment will be reduced if a greater number of samples can be multiplexed within a capture reaction. In the human genome, target enrichment of regions totaling 521 647 bp was captured by pre‐capture multiplexing of 12 and 16 samples, and it was observed that the average depth of coverage was significantly reduced in 16‐plex capturing compared with 12‐plex and nonmultiplexed capturing (Shearer *et al*., 2012). Because the cumulative length of our target regions was more than 80 times larger than in Shearer *et al*. (2012), we estimated that multiplexing of 12 or less samples would be suitable for our whole‐exome sequencing purposes. To test the capability of pre‐capture multiplexing for whole‐exome target enrichment in rice, we mixed an equal molar amount from eight libraries, and then performed target enrichment and sequencing to evaluate the performance of pre‐capture multiplexing in whole‐exome sequencing. The sequencing statistics for the eight mutants, including the 7‐3B mutant as a control, are presented in Table S4. When a similar amount (~6 billion bases) of sequencing data was obtained for each mutant, the total number of aligned reads/bases, and the fraction of the reads/bases mapped in and near the bait regions was comparable to individually captured samples and was not significantly affected by pre‐capture multiplexing. The ratio of ‘on bait bases’ to ‘near bait bases’ was significantly decreased from between 3.20 and 6.39 to between 1.90 and 2.19 in individual and multiplexed target capturing, respectively. This result indicated that more reads were derived from the borders and outside of the target regions, and caused lower read depth in each target region and overall mean target coverage (Tables S1 and S4). The proportion (percentage) of sequencing reads from each of the multiplexed samples was 12.50 ± 0.68 (average ± SD), which showed good agreement with the theoretical proportion for an equal molar mixture of eight samples. This result indicated that the difference in the index sequences and the subtle differences that existed in the sequence context did not affect the efficiency of target enrichment and sequencing. Although there is still the possibility that either higher or lower multiplexing during target capturing would give a better result, we concluded that the pre‐capture multiplexing of eight samples produces sufficient quality and quantity of target exon sequences. It is certain that multiplexing causes a significant decrease in the on‐target frequency when compared with the individually processed samples. Given that the sequencing cost is steadily decreasing over time, a substantial cost reduction in target capturing makes the overall sequencing cost more affordable, even though the on‐target frequency is compromised significantly.

### Characteristics of carbon‐ion beam‐induced mutations detected by massively parallel sequencing of unselected rice populations

Previous attempts to characterize heavy‐ion beam‐induced mutations were mostly based on the analysis of mutants that were selected on the basis of morphological features, therefore the resulting mutational characteristics may reflect the effects of mutant selections. Therefore, in the present study, we determined the characteristics of heavy‐ion‐induced mutations using the established multiplexed target exon‐enrichment sequencing technique in unselected M_2_ populations. These M_2_ plants were visually indistinguishable from non‐irradiated Nipponbare plants. We sampled an equal amount (by weight) of true leaf tissues from eight individual M_2_ plants in each line, mixed the samples, and subjected the resulting genomic DNA extracts to sequencing library preparation and target enrichment. This process equalizes the genetic differences among individual M_2_ progenies that inherited different sets of alleles from the same mutagenized M_1_ plant and minimizes the risk of bias on sample selection.

To identify the most effective irradiation dose, we first measured the survival rate at each irradiation dose in a greenhouse 4 weeks after planting in soil (Figure S2). The survival rate was defined as the percentage of plants that showed normal phyllotaxis and leaf numbers. The survival rate was comparable with non‐irradiated Nipponbare controls up to 200 Gy, and then showed a significant and severe decline at 250 and 300 Gy, respectively. Next, approximate tendencies were determined for the infertility rates and mutation frequencies, which were calculated from the percentage of morphological mutants in the M_2_ generation, from two independent experiments (Table S5). The infertility percentage, defined as the percentage of infertile spikelets in the main panicle of M_1_ plants that developed less than 50 filled seeds per panicle, increased to 22.2 and 24.3% with 175 and 200 Gy irradiation, respectively. The mutation frequency, which is the percentage of M_2_ lines with chlorophyll‐deficient mutants (CDM; albino, pale green, leaf yellowing, virescent, and striped phenotypes) observed in at least one plant, was highest in dry seeds with 175 Gy irradiation (14.6%), followed by 150 Gy (10.9%) and 200 Gy (8.8%). We further confirmed with an additional two independent populations that the infertility rate and mutation frequency at 150 and 175 Gy were in a comparable range (Experiment 2 in Table S5). From these results, we considered that 150 Gy irradiation of dry seeds was the optimum irradiation condition, because it showed one of the highest mutation frequencies while still maintaining satisfactory fertility. We analyzed 110 randomly chosen independent M_2_ lines (each line derived from a different M_1_ plant) for this irradiation condition. The sequencing coverage differed slightly among the samples, which may greatly affect coverage outside of the target exon regions. Therefore, we focused on the mutations located within the target regions that we aimed to capture by probe hybridizations.

The bioinformatics pipeline detected 997 mutations from the 110 independent M_2_ lines analyzed. The mutations consisted of 573 SNVs (57.5% of all mutations), 372 deletions (37.3%), 36 insertions (3.6%), 13 replacements (a type of mutation combining deletions and insertions; 1.3%), and three inversions (0.3%). Despite the previously reported characteristics that heavy‐ion beams mainly induced deletions in the plant genome, the most frequent type of mutations were SNVs followed by deletions. These two types of mutations accounted for 94.8% of all mutations, and the frequency of insertions, replacements, and inversions was approximately 10 times less than that of SNVs and deletions. These results indicated that carbon‐ion beam irradiation induces mostly SNVs and deletions in the rice genome. The properties in each type of induced mutation, such as the size distribution of deletions and insertions and the base‐change frequency in SNVs, are also important factors as a physical mutagen. The size distribution of deletions and insertions is summarized in Figure S2. Of the 372 deletion events, one‐third (124 events; 33.3%) was single‐nucleotide deletions and the remaining 199 events (53.4%) were sized between 2 and 10 bases in length (Figure S3A). Two large deletions, 10 005 and 624 508 bp, were also detected, but there is a strong possibility that the frequency of large deletions is underestimated due to the difficulty of target‐capture sequencing. Of the 36 insertion events, 23 (63.9%) were single‐nucleotide insertions and 12 events (33.3%) were between 2 and 11 bases in length. Only one event (31 bp insertion) was observed beyond that range among the 110 independent M_2_ lines analyzed (Figure S3B). Based on these observations, we interpreted that the size of deletions and insertions was relatively small, mostly spanning one or several bases in length, under the optimum condition of carbon‐ion beam irradiation (30 keV μm^−1^, 150 Gy) of dry seeds. The finding that insertions were distinctly less frequent than deletions suggests that the double‐stranded breaks of genomic DNA, caused by heavy‐ion particles, tend to be repaired cleanly without incorporation of filler DNA in rice.

**Figure 3:**
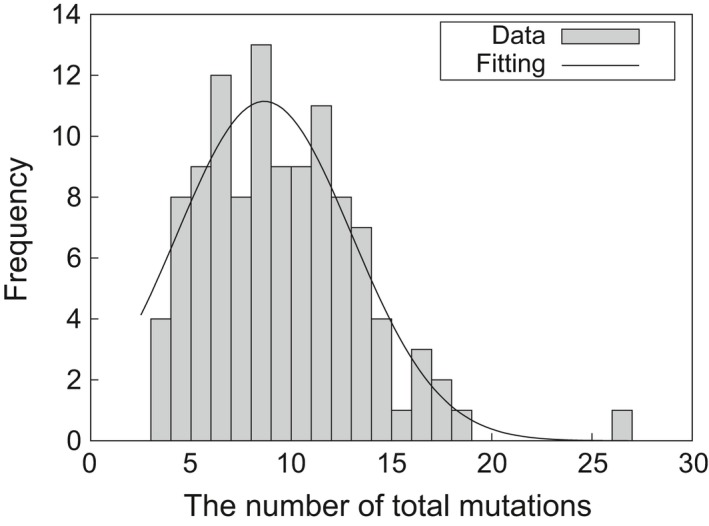
Distribution and Gaussian fit of the number of mutations per line The total number of mutations detected for each of 110 M_2_ lines derived from 150 Gy irradiation of dry seeds. On average 9.06 ± 0.37 (average ± SE) mutations were detected in an unselected M_2_ line. The solid line indicates goodness of fit to the Gaussian distribution (μ = 8.66, σ = 4.37).

**Figure 4:**
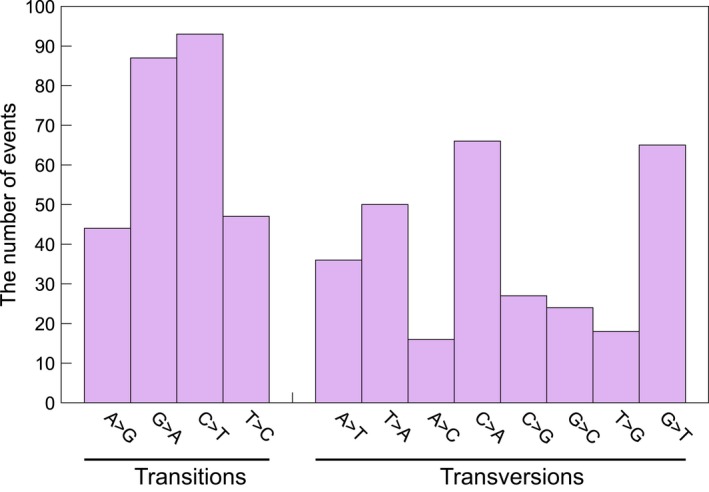
Nucleotide preference in single‐nucleotide mutations The number of transition and transversion events detected from the 110 M_2_ lines derived from 150 Gy irradiation of dry seeds for each combination of original and alternate bases. The total number of transition and transversion events was 271 and 302, respectively (ratio 1:1.07).

We analyzed the effect of the induced mutations in a sequence context. Of the 997 mutations detected, 504 events (50.6%) were predicted to alter the coding sequence of at least one exon, 169 events (17.0%) and 150 events (15.0%) were located in upstream regions (including 5′ untranslated regions) and downstream regions (including 3′ untranslated regions) of genes, respectively. The remaining mutations (174 events, 17.5%) were predicted to introduce synonymous change and occurred within introns. The distribution of the number of mutations per line and the goodness‐of‐fit to the Gaussian distribution is shown in Figure 3. On average, 9.06 ± 0.37 (average ± SE) mutations were induced in an independent M_2_ line following 150 Gy irradiation of dry seeds. The distribution was well represented by the Gaussian distribution (μ = 8.66, σ = 4.37). In the nucleotide context, the number of transitions and transversions in single‐nucleotide mutations were 271 (47.3%) and 302 (52.7%) in the 110 M_2_ lines, respectively (Figure 4). This result reflects that the highly accelerated carbon ions produced at the RIBF do not produce many hydroxyl radicals, which cause generation of 8‐hydoxy‐2′‐deoxyguanosine (8‐OHdG) within the cell and induce GA to CT transitions (Cheng *et al*., 1992). There were 96, 115, 176, and 186 single‐nucleotide mutation events at adenine, thymine, cytosine, and guanine nucleotides, respectively, and the AT to GC ratio at these mutated sites was 1:1.71. The AT to GC ratio of the entire target regions was 1:1.07, which indicated that guanine and cytosine nucleotides were approximately 1.60 times more prone to mutation than adenine and thymine nucleotides.

**Figure 5:**
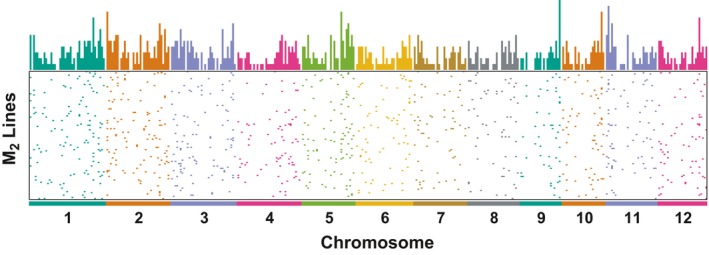
Chromosomal distribution and frequency of mutations The chromosomal location of all mutation events detected within the target exon regions for each M_2_ line derived from 150 Gy irradiation of dry seeds. The relative mutation frequency (per Mbp) is plotted above. The mutations were distributed on all chromosomes. Note that fewer target exon regions were subjected to target‐capture sequencing in the centromeric regions.

In theory, heavy‐ion beams cause entirely random mutations in the genome regardless of chromosome location and sequence context as the nature of physical mutagens. We tested this hypothesis with the sequence dataset for the 110 M_2_ lines. The chromosomal distribution and mutation frequency are illustrated in Figure 5 and summarized in Table S6. As expected, the mutations were distributed randomly among all chromosomes, except for the centromeric regions, which contain much fewer target regions compared with euchromatine regions. The total number of mutations within the target regions was indicated to be proportional to the total length of the target regions, regardless of the chromosome that the target region resides on. The average mutation frequency was 0.98 ± 0.16 (average ± SD) mutations per million bases of target regions per line. Surprisingly, the mutation frequency differed among the 12 chromosomes: the average mutation frequency per chromosome differed 1.56 times between the highest (chromosome 7; 0.77 mutations per million target bases per line) and the lowest (chromosome 11; 1.20 mutations per million target bases per line) frequencies. The mutation frequency may be influenced by the chromosomal location of genes essential for survival; however, it was not entirely clear because the number of mutations induced in each chromosome was relatively small in this particular dataset and may interfere with the results. From these observations, we concluded that mutagenesis by heavy‐ion beam is at least near random and shows a moderate preference for guanine and cytosine nucleotides. In addition, there might be sequence contexts in the rice genome, presumably essential genes, that prevent the transmission of a mutation to the next generation.

### A sequencing‐based indicator to determine the optimum irradiation condition for mutation induction

In principle, the number of mutations inducible in a genome is linearly correlated to irradiation dose, which defines the chance of mutations occurring. However, in practice, the maximum mutation frequency is observed at a certain irradiation dose, above which the mutation frequency and survival are decreased, presumably due to inactivation of genes essential for survival as well as cell‐cycle arrest owing to the DNA integrity checkpoint mechanisms (De Schutter *et al*., 2007). For practical mutation breeding, it is important to identify the optimal dose for mutation induction in each target organism because the size of the population required for screening is highly dependent on the mutation frequency. This is often achieved by determining the frequency of morphological mutants (e.g., proportion of CDMs) in the M_2_ generation. Although simple, this process is labor intensive and time consuming. Therefore, it is ideal if an indicator correlated with the optimized dose for mutation induction can be determined rapidly by massively parallel sequencing of unselected populations. For this particular purpose, we sequenced a small number of additional M_2_ populations (9–12 lines per condition) for different irradiation conditions (75, 100, 175, and 200 Gy irradiation of dry seeds and 15 Gy irradiation of imbibed seeds) and eight pools of non‐irradiated Nipponbare as the control, using the same method described above for the 110 M_2_ lines following 150 Gy irradiation of dry seeds.

**Figure 6:**
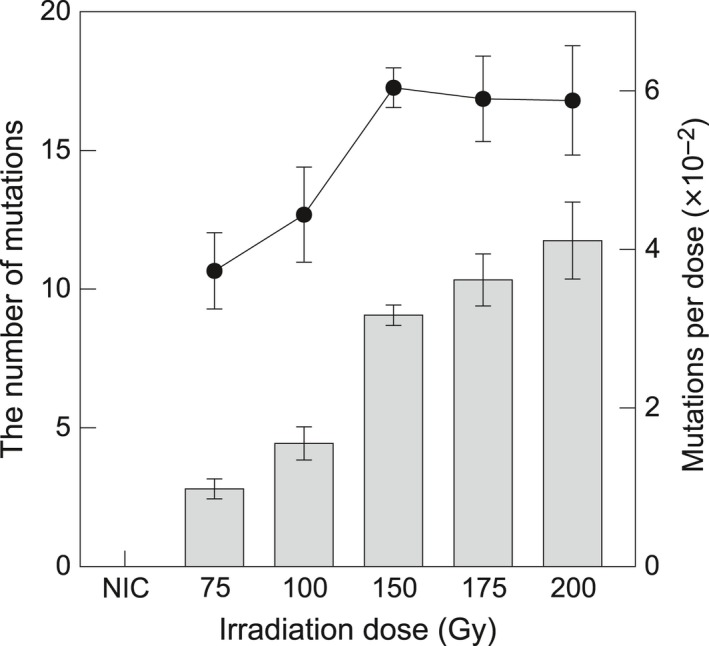
Number of line‐specific mutations in heavy‐ion‐irradiated, unselected Nipponbare populations The mean number of line‐specific mutations (bars) for populations subjected to different irradiation doses (15, 75, 100 and 150 Gy), linear energy transfer (30 and 50 keV μm^−1^), and seed conditions (dry and imbibed seeds). The total number of mutations per irradiation dose is indicated by solid circles. Error bars represent the standard error. NIC, non‐irradiated control.

Figure 6 shows the overall numbers of mutations detected by the pipeline for each condition. The number of mutations induced within the target exon regions per line increased with the irradiation dose at least up to 200 Gy; 2.80 ± 0.36 (average ± SD), 4.44 ± 0.60, 10.33 ± 0.94, and 11.75 ± 1.39 mutations were detected in the M_2_ generation following 75, 100, 175, and 200 Gy irradiation of dry seeds, respectively (Table 2). In the case of irradiation of imbibed seeds, 2.75 ± 0.63 (average ± SE) mutations were detected in the M_2_ generation following 15 Gy irradiation. This number corresponded to that between 75 and 100 Gy irradiation of dry seeds, which indicated that the increase in radiation sensitivity of imbibed seeds is attributable to the frequency of mutations introduced into the genome. Interestingly, the percentage of deletions and insertions was highest following 15 Gy irradiation of imbibed seeds (46.9%). This finding may indicate a correlation between the physical state of the chromosome and/or higher LET (50 keV μm^−1^) and the frequency of induced mutations. No mutation was detected within the target regions for all non‐irradiated Nipponbare control pools, which indicated that the innate mutation frequency was below the detection limit in this particular analysis.

**Table 2:** Survival and mutation frequencies in the M_2_ generation

Seed condition	Dose (Gy)	LET (keV/um)	Num. of M_1_ lines harvested	Survival rate (%)	Fertility (%)	Mutation rate (%)	Number of lines with CDMs				
							Albino	Palegreen	Yellow	Virescent	Stripe
Dry	0	—	98	98	100	0	0	0	0	0	0
Dry	75	30	95	95	100	6.3	3	3	0	0	0
Dry	100	30	97	97	100	2.1	1	1	0	0	0
Dry	150	30	243	81	93.7	9.9	10	7	3	2	2
Dry	175	30	225	75	80.3	15.6	15	8	2	4	6
Dry	200	30	97	97	65.0	8.3	4	3	0	1	0
Wet	15	50	226	75	n.d.	6.6	6	2	4	0	3

Survival, which is the percentage of the M_1_ lines grown from the irradiated seeds in a paddy field, was comparable with that of the non‐irradiated Nipponbare control up to 100 Gy irradiation (95–97% survival compared with the non‐irradiated control). Significant decline in survival was observed for 150 Gy (81%) and 175 Gy (75%) irradiation of dry seeds. n.d., not determined.

Contrary to the physical expectation that the total number of mutations induced per line changes in parallel with the irradiation dose, as a higher dose confers greater energy to cause DNA lesions, the number of mutations per irradiation dose showed a plateau at or above 150 Gy (Figure 6). The number of mutations per irradiation dose per line were (3.73 ± 0.48) × 10^−2^, (4.44 ± 0.60) × 10^−2^, (6.04 ± 0.25) × 10^−2^, (5.90 ± 0.54) × 10^−2^, and (5.88 ± 0.69) × 10^−2^ per Gy for 75, 100, 150, 175, and 200 Gy irradiation of dry seeds, respectively. The corresponding value was (18.33 ± 4.19) × 10^−2^ per Gy for 15 Gy irradiation of imbibed seeds. The fact that the mutation efficiency was highest following 150 Gy irradiation of dry seeds indicated that mutation frequency more‐or‐less reflected the balance between the introduction of mutations into the genome and survival. In addition, mutation frequency may be a useful indicator for sequencing‐based and rapid determination of the optimum irradiation condition without the need for labor‐intensive isolation of mutants.

## Discussion

Low‐LET ionizing radiation, such as X‐rays and γ‐rays, have been widely used for mutation breeding over the past nine decades. It is well known that high‐LET ionizing radiation, including accelerated heavy‐ion particles, induce different types of mutations compared with those generated by low‐LET radiations, by introducing clustered damage and causing double‐stranded breaks to DNA (Ward, 1994; Goodhead, 1999). High‐LET radiation has been introduced relatively recently for mutagenesis, therefore one can expect to obtain novel mutants that have not been identified previously from a population mutagenized with low‐LET radiation and other commonly used mutagens. In the present study, we established a whole exon enrichment sequencing procedure in rice using a custom‐designed commercial target enrichment system. Target exon enrichment shrinks the required sequencing depth by approximately one‐fifth compared with WGS. Therefore, by employing whole exon enrichment sequencing, five‐times more samples can be analyzed at simultaneously for the same sequencing cost. Although the exon enrichment probes and associated hybridization and recovery reagents constitute a substantial percentage of the sequencing costs, one can linearly reduce the cost per sample by employing pre‐capture sample multiplexing. We multiplexed eight samples for target capture, and then combined two capture reactions and sequenced the samples in one lane on an Illumina HiSeq 4000 system, which yielded approximately 3.5 Gb (more than 80‐times the equivalent of the target regions) of clean sequences per sample. We noticed that the reads that corresponded to the flanks of target regions and repeated sequences tended to remain after target enrichment and caused a lower on‐target frequency when pre‐capture sample multiplexing was applied. Given that we observed better performance in capture specificity when we used a rice repetitive DNA fragment library instead of a commercial universal blocking reagent, the hybridization specificity would be improved if an excess amount of unlabeled DNA fragments that correspond to the flanks of the target regions and repetitive sequences was added to the hybridization reaction. Our sequence datasets should be useful to determine such sequences for further improvement in the performance of hybridization‐based rice exon enrichment and to minimize the cost, because data were acquired from over 100 samples treated identically throughout the process.

Massively parallel sequencing‐based identification provides outstanding resolution and sensitivity for the determination of mutations. One problem we encountered in the initial analysis was that there is an unrealistic number (often over 10 000) of mutations listed in the final outputs of the GATK and Pindel programs. A filtering strategy that is similar to the one proposed for Arabidopsis (Ishii *et al*., 2016) was employed and worked well to exclude intracultivar variations that constituted more than 99% of the mutations listed, and showed good agreement with the results of capillary dideoxy sequencing of PCR fragments amplified with flanking primers. Although rice is self‐fertilizing, we realize there is a significant chance of outcrossing, especially in a mutant with reduced fertility but far less often in a carbon‐ion beam‐irradiated, unselected M_2_ population (only one possible contaminant was detected in 166 M_2_ lines). Therefore, one should show extra caution when propagating and analyzing mutants with a visible phenotype. As a solution, we are currently employing pre‐screening with simple sequence repeat markers that can distinguish *japonica* and *indica* rice cultivars (Ebana *et al*., 2008), before proceeding to library preparation and target enrichment, when analyzing such mutants.

In the present study, we determined the frequency and physical distribution of mutations caused by highly accelerated carbon‐ion beams in unselected M_2_ progenies. The data indicated that carbon‐ion beam irradiation caused an average of 9.06 ± 0.37 (average ± SE) mutations in the capture target exon regions under the optimum irradiation condition (dry seeds, 13% water content, 30 keV μm^−1^, 150 Gy), whereas no mutations were detected within the capture target regions in non‐irradiated Nipponbare plants. Based on a linear interpolation from the proportion of target exon regions against the entire genome (34 040 553 bases of target exon regions within 373 245 519 bases of the rice genome), 10.96 times more mutations are expected per genome when the mutation frequency is postulated to be identical between the target exon regions and the remaining genomic regions. The theoretical number of mutations per genome is 30.7, 48.7, 99.3, 113.2, and 128.8 mutations for 75, 100, 150, 175, and 200 Gy irradiation of dry seeds, respectively, and 30.1 for 15 Gy irradiation of imbibed seeds. Outside of the target exon regions are mostly intergenic and repeated regions, therefore the actual number of mutations per genome is higher than the above‐mentioned numbers, because mutations in intergenic and repeated regions have much less chance to inactivate genes that are necessary for survival and reproduction compared with mutations in protein‐coding exons. The above‐mentioned frequencies are roughly one order of magnitude less than that reported in rice seeds treated with the alkylating agent ethyl methanesulfonate (EMS; Henry *et al*., 2014), which indicates that heavy‐ion beam irradiation introduces much fewer mutations compared with EMS and presumably other chemical mutagens that achieve a similar mutation frequency to that of EMS. This difference reflects the favorable property of heavy‐ion beams in mutation breeding: the phenotypes of mutant plants can be fixed at an earlier stage during the development process, therefore it has been utilized as the preferred mutagen for the breeding of crops and other agricultural plant species.

For fast‐neutron irradiation to X.Kitaake, a transgenic derivative of the *japonica* rice cultivar ‘Kitaake’, the number of mutations introduced per genome ranged from 28 to 78 in each mutagenized M_3_ line, with an average of 59, following 20 Gy irradiation of seeds (Li *et al*., 2016a). This mutation frequency was comparable to the above‐mentioned theoretical number of mutations following 100 Gy irradiation of dry seeds, and may indicate that carbon‐ion beams affect survival to a lesser degree and mutations may accumulate with increasingly higher doses. Li *et al*. (2016a) also reported that the size of fast‐neutron‐induced insertions were relatively small: 73.2% of all insertions and deletions were equal or less than 10 bp as assessed by WGS. In the case of carbon‐ion beams, 74.8% of all insertions and deletions were ≤6 bp, and 87.5% were ≤10 bp. Although this comparison may show a potential systematic bias between WGS and targeted exome sequencing, the fundamental characteristics of the mutations induced by fast neutrons and carbon‐ion beams would be similar in rice in this particular dataset. However, the pattern of SNVs clearly differs between carbon‐ion beams and γ rays. We observed a relatively lower frequency of GA to TC transitions and a higher frequency of CG to GT transversions, and GA to TC transitions were almost or more than twice as frequent in the M_2_ progenies from γ‐ray‐induced mutants (Li *et al*., 2016b). The GA to TC transitions are due to oxidative reactions by hydroxyl radicals (Cheng *et al*., 1992). More than half of the biological effect of low‐LET ionizing radiation, including γ‐rays, is the result of indirect radiation mostly caused by hydroxyl radicals (Lagoda, 2012).

It should be noted that some mutations might be undetectable owing to the nature of target‐enriched sequencing (uneven and discontinuous coverage), particularly large deletions and chromosome rearrangements. Target coverage‐based detection could not be applied in the present study because multiple M_2_ progenies were pooled in each line to equalize the genotypes that may differ among individual plants, therefore all mutations were detected as heterozygous. Consequently, a robust algorithm that can distinguish the difference in read depth between heterozygous and homozygous genotypes is needed to detect large deletions from target‐enriched sequencing results. Identification of copy number variations from whole‐exome datasets is of considerable interest and numerous algorithms have been proposed. Of these algorithms, we applied XHMM (Fromer *et al*., 2012), which utilizes a hidden Markov model for detection, to the 80 samples in the whole‐exome dataset. The program returned 53 possible copy number variations (duplications and deletions) in the genome. We visually inspected all of these possible copy number variation (CNV) sites on a read alignment viewer, but none of these seemed likely to be present and appeared to represent false positives (Table S7). Therefore, further improvements in both target enrichment and the sequencing process and in the algorithm for mutation detection are required to enable more comprehensive detection of the types of mutations that are difficult to detect. The 165 carbon‐ion beam‐irradiated M_2_ lines sequenced in the present study will provide a valuable resource for screening projects in rice. Using the line‐specific mutations that we identified using the bioinformatics pipeline, these sequenced lines as well as the established exome sequencing and bioinformatics procedure will also be useful resources for reverse‐genetic approaches.

## Experimental procedures

### Heavy‐ion beam irradiation and plant growth

Rice ‘Nipponbare’ seeds were placed in a temperature‐ and humidity‐controlled incubator (maintained at 22°C and 70% relative humidity; Yamato Scientific, Tokyo, Japan) to adjust the water content to 13% using a grain moisture tester (Riceter F2, Kett Electric Laboratory, Tokyo, Japan). For dry seed irradiation, the seeds were placed in a polypropylene/nylon bag (Hybri‐Bag Hard, Cosmo Bio, Tokyo, Japan) 1 day before irradiation. For imbibed seed irradiation, the seeds were soaked in sterilized distilled water for 3 days at 28°C in the dark, and then packed in a bag as described above. Carbon (^12^C^6+^) ions were accelerated to 135 MeV nucleon^−1^ by an azimuthally varying field cyclotron and the RIKEN Ring Cyclotron accelerators at the RIBF. The irradiation experiments were performed at the E5B beamline at the RIBF. The rice seeds were irradiated with the accelerated carbon ions at the defined dose and LET using an automated irradiation system (Ryuto *et al*., 2008).

The irradiated and non‐irradiated control seeds (M_1_ seeds) were germinated and grown individually under standard agricultural practices in a paddy field. The M_2_ seeds were harvested from each plant, and the fertility rate and mutation rate were determined. The fertility rate, defined as the percentage of the spikelets that were fertile (at least 50 seeds filled) in the panicle on the primary branch of M_1_ plants. The mutation rate was calculated based on the number of M_1_ lines that showed CDMs in the M_2_ generation. For exome sequencing analysis, 10–20 M_2_ seeds from each M_1_ line were germinated and grown, then equal amounts of leaf tissues were collected from eight plants and mixed together to provide comprehensive coverage of the mutations induced by heavy‐ion beam irradiation in each line, regardless of the genetic segregation pattern and resulting zygosity. In total, 100 mg leaf tissue was used to extract genomic DNA for each line using the MagExtractor^®^ ‐Plant Genome‐ kit (Toyobo, Kyoto, Japan). The quality of the purified DNA was determined by spectrometry and agarose gel electrophoresis. The DNA quantity was determined fluorometrically using the Qubit^®^ dsDNA Assay Kit (Life Technologies, Carlsbad, CA, USA).

### Whole exon enrichment and massively parallel sequencing

Purified DNA (1 μg) was subjected to fragmentation by sonication at the target length of 300 bp using a Covaris S220 ultrasonicator (Covaris, Woburn, MA, USA). The sequencing libraries were prepared using the NEXTflex™ Rapid DNA‐Seq Kit (Bioo Scientific, Austin, TX, USA) in combination with SeqCap Adapter Kits (Roche Diagnostics, Mannheim, Germany) in accordance with the manufacturer's instructions. Custom‐designed rice whole exon enrichment probes (SeqCap developer library, Roche Diagnostics) were based on the Nipponbare reference genome IRGSP Build 5 and the corresponding exon and intron coordinates. All exons and 50 bases each of both flanking regions were subjected for probe design, except for the one annotated as transposase. The exon enrichment probes (SeqCap EZ developer library, Roche Diagnostics) were designed and synthesized by the company's proprietary method. An equal molar mixture of eight libraries was prepared for each exon enrichment, and subjected to hybridization with the exon enrichment probes. An aliquot (4.5 μl) of the target exon enrichment probes was hybridized to a mixed library (1 μg) at 47°C for 72 h, then the probe‐library hybrids were captured and recovered in accordance with the SeqCap EZ Library SR User's Guide Version 5.1 (Roche Diagnostics). Successful exon enrichment was monitored by real‐time quantitative PCR and assessment of the fragment size distribution using an Agilent 2100 Bioanalyzer (Agilent Technologies, Santa Clara, CA, USA). The resulting exon‐enriched libraries were subjected to sequencing on HiSeq™ 2500 and 4000 instruments (Illumina, San Diego, CA, USA).

### Bioinformatics

An in‐house bioinformatics pipeline was implemented on the Hokusai GreatWave system, a massively parallel computing platform operated by the RIKEN Advanced Center for Computing and Communication. The clean sequencing reads were mapped to the reference Nipponbare IRGSP‐1.0 sequences using the Burrows−Wheeler Aligner (BWA) software (Li and Durbin, 2009), then converted to the standard BAM format, sorted, duplicated reads were marked, realigned near insertions and deletions, and quality scores recalibrated using a combination of Samtools (Li *et al*., 2009), Picard (Broad Institute, 2017), and GATK (McKenna *et al*., 2010). Variant callings were done using a combination of the ‘Unified Genotyper’ function in GATK, Pindel (Ye *et al*., 2009), and Bedtools (Quinlan and Hall, 2010) with the default settings. The called variants were annotated using the SnpEff program (Cingolani *et al*., 2012) to assess the possible effect of the variants on gene functions, based on the gene structure predictions in IRGSP‐1.0 annotations (IRGSP‐1.0_representative, accessed 2015/3/31). Four or more mutants were subjected to simultaneous variant calling, and the variants that were shared between two or more mutants were filtered out as they were intracultivar variations that already existed in the parental Nipponbare line used. In the case of variants identified by GATK and Pindel, variants that were supported by 10 or more mapped reads were considered significant. After combining all line‐specific mutations from the three variant calling programs and removing duplicated records, variants that were located within the target regions were extracted using the ‘intersect’ tool in Bedtools. In parallel with mapping reads and variant calling, the quality of input sequencing reads was checked using the FastQC program (Andrews, 2010), and the results were stored as an HTML‐formatted report for review. The entire process was executed automatically and in parallel through a set of shell scripts and a batch job controlling system. The developed filtering program, including line‐specificity determination described above, were implemented in the C++ programming language on a Linux platform. The source codes and detailed usage instructions are freely available from GitHub (https://github.com/ion-beam-breeding/RiceExome) under GNU GPL version 2.

## Author Contributions

HI and TA conceived and designed the experiments. HI, RM, YS, YH, and TA performed the experiments. HI, RM and TA analyzed the data. HI wrote the paper. All authors read and approved the final manuscript.

## Conflict of Interest

The authors declare that there are no conflicts of interest.

## Supporting information


**Figure S1.** Confirmation of a large (102 158 bp) deletion in the 6‐62 mutant.

**Figure S2.** Determination of survival rate with different irradiation dose.

**Figure S3.** Size distribution of deletions and insertions in an unselected carbon‐ion beam‐irradiated rice population.

**Table S1.** Sequencing statistics for individually captured carbon‐ and neon‐ion beam‐induced mutants (ordered by LET).

**Table S2.** Verification of the detected variants by PCR and Sanger sequencing.

**Table S3.** Covered bases (‘Covered’), average read depth (‘Average depth’), and the fraction of covered bases against the target region (‘Frac.’) in the 6‐62 mutant and the average of three mutants (3‐14, 7‐30, and 7‐3B).

**Table S4.** Sequencing statistics for eight mutants for pre‐multiplexed target enrichment.

**Table S5.** Survival and mutation frequencies in the M_2_ generation after irradiation of carbon‐ion beam (LET: 23–30 keV μm^−1^) to dry seeds.

**Table S6.** Chromosomal distribution of mutations in M_2_ progenies following 150 Gy irradiation of dry seeds.

**Table S7.** XHMM output and their visual inspection results.

**Data S1.** List of target exons in IRGSP Build 5 and Os‐Nipponbare‐Reference‐IRGSP‐1.0 sequences.

## Data Availability

The high‐throughput sequencing reads obtained from irradiated and non‐irradiated lines were deposited in the Sequence Read Archive under the accession number SRP145251. All other relevant data are within the paper and the supplemental files. Program source codes and a user manual will be freely available through GitHub (https://github.com/ion-beam-breeding/RiceExome) under GNU General Public License (GPL) version 2, upon acceptance of this paper.
