# Supplementary material for: Targeted exome sequencing of unselected heavy‐ion beam‐irradiated populations reveals less‐biased mutation characteristics in the rice genome
Source: Plant J. 2019 Feb 25;98(2):301–14. doi: 10.1111/tpj.14213 (PMC6850588; doi:10.1111/tpj.14213)
Supplement: Supplementary file 1 — Figure S1. Confirmation of a large (102 158 bp) deletion in the 6‐62 mutant. [file TPJ-98-301-s001.pdf]

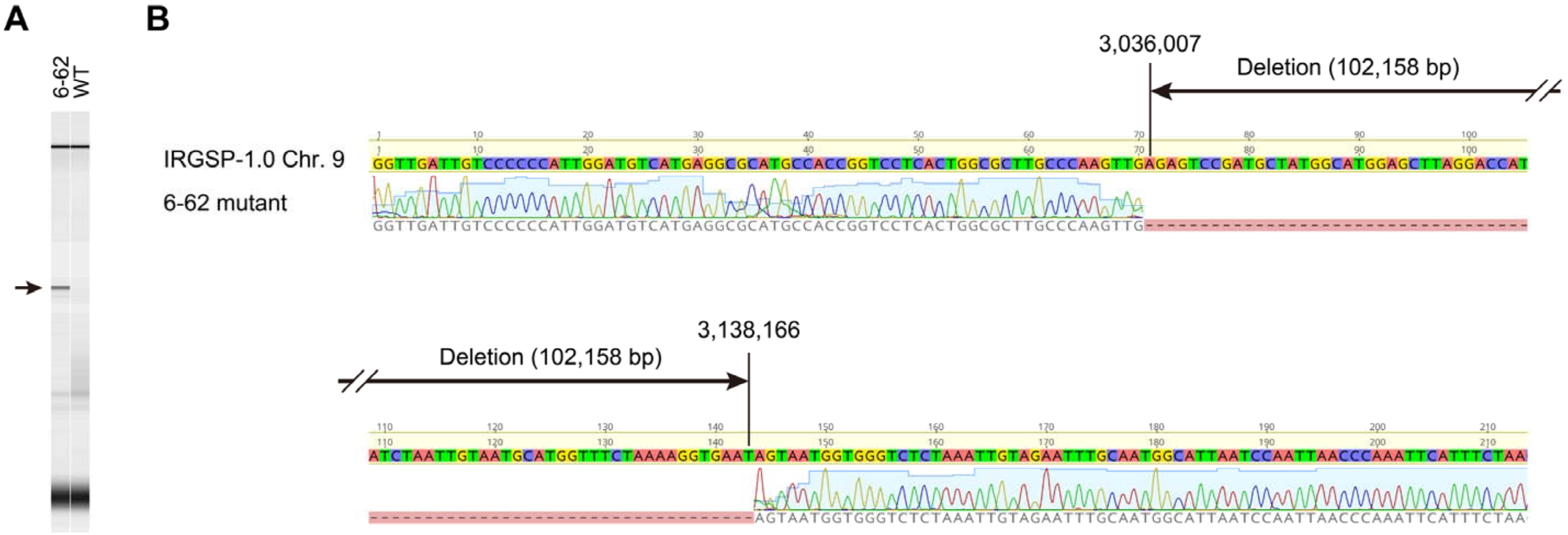

Figure S1. Confirmation of a large (102,158 bp) deletion in the 6-62 mutant

A: a pair of PCR primers were designed upstream and downstream of the deletion, and subjected for amplification from genomic DNA from the 6-62 mutant and wild-type Nipponbare. The arrow indicates the 303 bp band expected in the 6-62 mutant. B: A schematic drawing of the structure and flanks of the deletion. Comparison between PCR-direct sequencing of the amplified DNA fragment and the reference Nipponbare genomic sequence indicated that the deletion spans 102,158 bp in length and has no filler sequence.
