# Supplementary material for: Targeted exome sequencing of unselected heavy‐ion beam‐irradiated populations reveals less‐biased mutation characteristics in the rice genome
Source: Plant J. 2019 Feb 25;98(2):301–14. doi: 10.1111/tpj.14213 (PMC6850588; doi:10.1111/tpj.14213)
Supplement: Supplementary file 2 — Figure S2. Determination of survival rate with different irradiation dose. [file TPJ-98-301-s002.pdf]

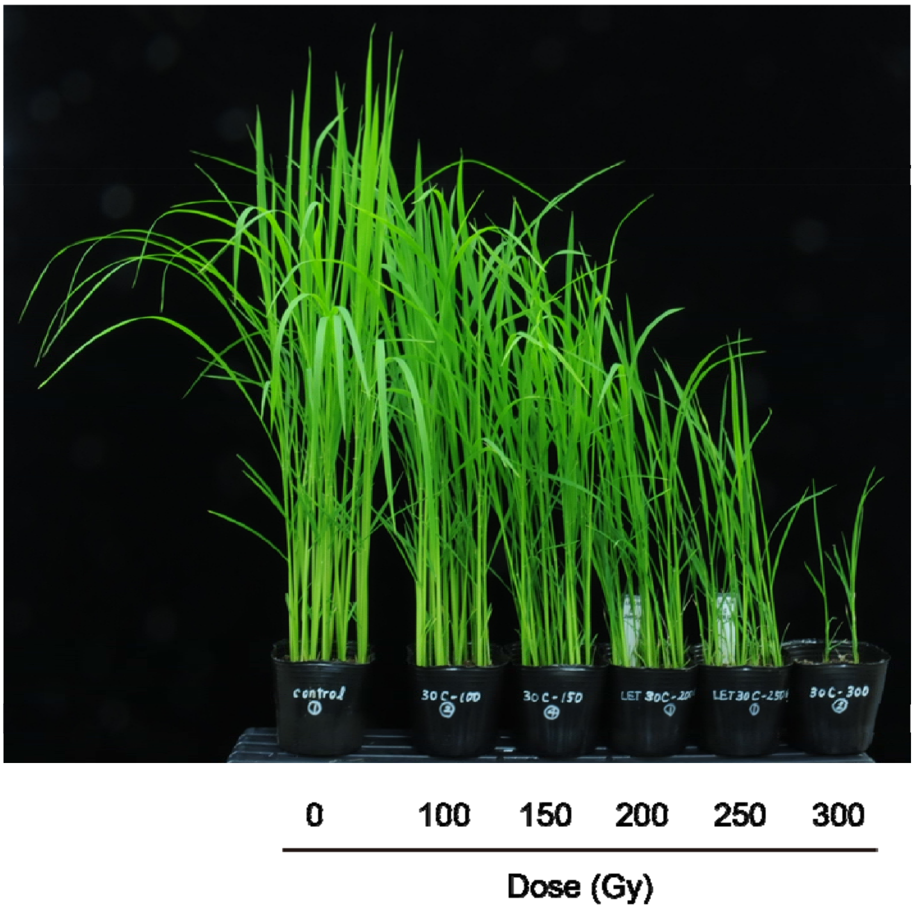

Figure S2. Determination of survival rate at different irradiation doses.

The survival rate, defined as the percentage of plants that showed normal phyllotaxis and leaf numbers, was determined in a greenhouse 4 weeks after sowing. The assay was conducted in triplicate with 100 seeds in each replicate, and a representative result is shown.
