# Supplementary material for: Targeted exome sequencing of unselected heavy‐ion beam‐irradiated populations reveals less‐biased mutation characteristics in the rice genome
Source: Plant J. 2019 Feb 25;98(2):301–14. doi: 10.1111/tpj.14213 (PMC6850588; doi:10.1111/tpj.14213)
Supplement: Supplementary file 3 — Figure S3. Size distribution of deletions and insertions in an unselected carbon‐ion beam‐irradiated rice population. [file TPJ-98-301-s003.pdf]

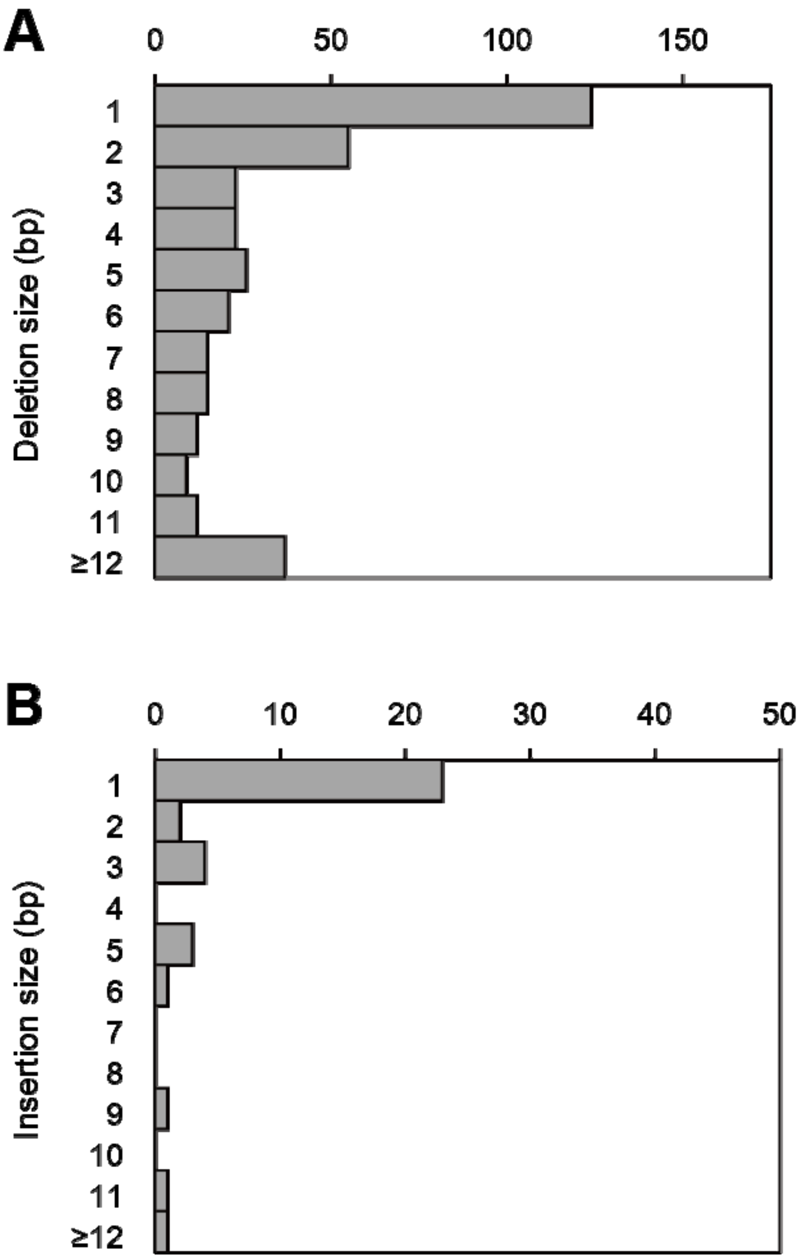


**Figure S3. Size distribution of deletions and insertions in an unselected carbon-ion beam-irradiated rice population**

The size distribution of deletions (A) and insertions (B) observed in 110 independent sequenced $M_2$ lines derived from 150 Gy irradiation of dry seeds. The majority of the detected insertion/deletion mutations involved a single nucleotide or several nucleotides. The frequency of insertions was approximately 10 times lower than that of deletions.
