# Supplementary material for: Targeted exome sequencing of unselected heavy‐ion beam‐irradiated populations reveals less‐biased mutation characteristics in the rice genome
Source: Plant J. 2019 Feb 25;98(2):301–14. doi: 10.1111/tpj.14213 (PMC6850588; doi:10.1111/tpj.14213)
Supplement: Supplementary file 4 — Table S1. Sequencing statistics for individually captured carbon‐ and neon‐ion beam‐induced mutants (ordered by LET). [file TPJ-98-301-s004.pdf]

Table S1. Sequencing statistics for individually captured carbon- and neon-ion beam-induced mutants (ordered by LET)

Line	6-99	5-24	13-45	3-14	3-51	5-12
Background	Nipponbare	Nipponbare	Nipponbare	Nipponbare	Nipponbare	Nipponbare
Irradiation condition [*1]	Carbon, 40 Gy 23 keV/µm	Carbon, 20 Gy 30 keV/µm	Carbon, 20 Gy 30 keV/µm	Carbon, 15 Gy 50 keV/µm	Carbon, 15 Gy 50 keV/µm	Carbon, 15 Gy 50 keV/µm
Phenotype	Salt tolerance	Late maturing	Ripening failure	Ripening failure	Dwarf	Stripe
Blocking DNA[*2]	SeqCap	SeqCap	SeqCap	Custom	SeqCap	Custom
Total read bases	9,982,229,800	7,354,545,000	6,481,829,200	5,637,744,900	6,289,462,100	6,270,056,100
Fold target	293.25	216.05	190.41	165.62	184.76	184.19
Total reads aligned	99,795,562	73,318,867	64,728,733	56,166,726	62,151,510	62,486,773
Reads aligned (%)	99.97	99.69	99.86	99.63	98.82	99.66
Total bases aligned	9,967,154,660	7,298,079,473	6,460,553,606	5,599,303,871	6,141,965,753	6,223,463,364
On bait bases	7,510,416,096	4,846,015,250	4,893,906,840	3,454,164,026	3,681,465,976	4,067,529,539
Near bait bases	1,204,980,454	1,381,281,118	813,161,469	1,084,555,821	1,260,015,538	1,256,653,845
% on/near bait	87.44	85.33	88.34	81.06	80.45	85.55
Mean target coverage	127.74	96.53	86.73	71.57	69.15	80.32
% target read > 1x	97.36	95.27	96.05	96.71	94.52	94.34
% target read > 10x	88.77	84.74	88.73	86.76	82.07	82.06
% target read > 20x	84.65	79.68	84.20	80.53	75.17	76.09
% target read > 30x	80.26	75.73	78.98	74.83	69.82	71.44
% target read > 50x	72.17	68.08	68.38	61.45	59.51	62.61

Table S1. (continued)

Line	4-13	18-36	6-62	7-3B	Ne-1779	7-30	14-45
Background	Nipponbare	Nipponbare	Nipponbare	Nipponbare	Nipponbare	Nipponbare	Unknown (outcrossed)
Irradiation condition *1	Carbon, 15 Gy 60 keV/μm	Carbon, 15 Gy 60 keV/μm	Neon, 15 Gy 63 keV/μm	Neon, 10 Gy 63 keV/μm	Neon, 15 Gy 63 keV/μm	Neon, 15 Gy 70 keV/μm	Carbon, 20 Gy 40 keV/μm
Phenotype	Weak growth	Salt tolerance	Dwarf	Lesion mimic	Virescent	Pale green	Salt tolerant
Blocking DNA*2	Custom	SeqCap	Custom	Custom	SeqCap	Custom	Custom
Total read bases	7,195,028,700	9,556,373,300	5,955,444,100	6,077,810,500	6,345,199,400	7,275,933,700	9,665,989,800
Fold target	211.37	280.73	174.95	178.55	186.40	213.74	283.96
Total reads aligned	71,689,912	95,537,610	58,932,688	60,467,123	63,319,725	72,543,245	96,618,923
Reads aligned (%)	99.64	99.97	98.96	99.49	99.79	99.70	99.96
Total bases aligned	7,133,685,392	9,541,675,913	5,868,415,678	6,026,315,570	6,309,259,147	7,227,951,637	9,644,842,979
On bait bases	4,709,328,265	7,165,993,106	3,509,300,891	3,766,988,660	4,615,720,506	4,434,909,206	7,226,624,327
Near bait bases	1,340,676,976	1,152,232,803	1,094,982,673	1,167,510,170	998,009,006	1,345,236,775	1,131,073,073
% on/near bait	84.81	87.18	78.46	81.88	88.98	79.97	86.65
Mean target coverage	94.87	121.15	73.83	79.42	91.33	94.10	121.52
% target read > 1x	95.69	97.32	97.60	96.88	91.44	97.74	97.28
% target read > 10x	85.08	88.37	88.82	88.10	79.21	89.79	88.23
% target read > 20x	79.91	83.57	82.81	82.65	74.34	84.77	83.78
% target read > 30x	75.77	78.67	77.03	77.58	70.55	80.34	79.13
% target read > 50x	67.56	70.17	62.83	65.54	63.48	70.39	70.75

*1 Ion (cabon or neon), dose (Gy) and linear energy transfer (keV/μm)

*2 Custom: custom rice repetitive element (Cot-1) library; SeqCap: SeqCap EZ developer reagent
