# Supplementary material for: Targeted exome sequencing of unselected heavy‐ion beam‐irradiated populations reveals less‐biased mutation characteristics in the rice genome
Source: Plant J. 2019 Feb 25;98(2):301–14. doi: 10.1111/tpj.14213 (PMC6850588; doi:10.1111/tpj.14213)
Supplement: Supplementary file 6 — Table S3. Covered bases (‘Covered’), average read depth (‘Average depth’), and the fraction of covered bases against the target region (‘Frac.’) in the 6‐62 mutant and the average of three mutants (3‐14, 7‐30, and 7‐3B). [file TPJ-98-301-s006.pdf]

Table S3. Covered bases (“Covered”), average read depth (“Average depth”), and the fraction of covered bases against the target region (“Frac.”) in the 6-62 mutant and the average of three mutants (3-14, 7-30, and 7-3B)

Location			6-62 mutant			Average of 3 other mutants (3-14, 7-30, 7-3B)		
Chr.	Start	End	Covered	Average depth	Frac.	Covered	Average depth	Frac.
9	3,063,815	3,064,438	0	0	0	624	36.4	1
9	3,074,231	3,074,518	0	0	0	288	66.7	1
9	3,075,476	3,075,524	0	0	0	49	68.2	1
9	3,075,624	3,075,829	0	0	0	206	104.2	1
9	3,077,552	3,077,720	0	0	0	169	80.0	1
9	3,077,930	3,077,995	0	0	0	66	48.1	1
9	3,078,118	3,078,225	0	0	0	108	57.7	1
9	3,079,315	3,079,422	0	0	0	108	98.4	1
9	3,079,501	3,079,566	0	0	0	66	101.3	1
9	3,083,592	3,083,652	0	0	0	61	77.7	1
9	3,083,780	3,083,907	0	0	0	128	132.5	1
9	3,084,904	3,084,945	0	0	0	42	73.7	1
9	3,085,262	3,085,378	0	0	0	117	124.0	1
9	3,085,468	3,085,519	0	0	0	52	97.8	1
9	3,091,500	3,091,681	0	0	0	182	69.8	1
9	3,091,781	3,091,866	0	0	0	86	81.9	1
9	3,092,019	3,092,095	0	0	0	77	98.8	1
9	3,092,192	3,092,503	0	0	0	312	91.8	1
9	3,105,019	3,105,495	0	0	0	477	61.0	1
9	3,120,346	3,121,350	0	0	0	1005	157.3	1
9	3,120,435	3,120,887	0	0	0	453	180.8	1
9	3,121,449	3,121,625	0	0	0	177	122.4	1
9	3,121,723	3,121,836	0	0	0	114	112.1	1
9	3,122,802	3,122,906	0	0	0	105	198.5	1
9	3,123,118	3,123,252	0	0	0	135	82.4	1
9	3,123,336	3,123,391	0	0	0	56	60.9	1
9	3,123,703	3,123,819	0	0	0	117	105.3	1
9	3,124,103	3,124,233	0	0	0	131	63.8	1
9	3,124,352	3,124,915	0	0	0	564	79.4	1
9	3,126,807	3,127,160	0	0	0	354	51.1	1
