# Supplementary material for: Targeted exome sequencing of unselected heavy‐ion beam‐irradiated populations reveals less‐biased mutation characteristics in the rice genome
Source: Plant J. 2019 Feb 25;98(2):301–14. doi: 10.1111/tpj.14213 (PMC6850588; doi:10.1111/tpj.14213)
Supplement: Supplementary file 7 — Table S4. Sequencing statistics for eight mutants for pre‐multiplexed target enrichment. [file TPJ-98-301-s007.pdf]

Table S4. Sequencing statistics for eight mutants for pre-multiplexed target enrichment

| Line | 5-24 | 7-3B | D14-03 | D14-13 | D14-15 | D14-27 | D14-39 | D14-48 |
|---|---|---|---|---|---|---|---|---|
| Total read bases | 6,175,695,800 | 6,265,268,000 | 6,595,272,300 | 5,778,349,800 | 6,110,537,900 | 6,494,204,700 | 6,769,304,200 | 5,921,027,700 |
| Fold target | 181.42 | 184.05 | 193.75 | 169.75 | 179.51 | 190.78 | 198.86 | 173.94 |
| Total reads aligned | 61,381,532 | 62,315,604 | 65,531,678 | 57,459,155 | 60,708,668 | 64,602,576 | 67,242,840 | 58,925,509 |
| % aligned | 99.39 | 99.46 | 99.36 | 99.44 | 99.35 | 99.48 | 99.33 | 99.52 |
| Total bases aligned | 6,096,723,407 | 6,193,535,506 | 6,506,873,176 | 5,708,159,354 | 6,027,282,394 | 6,423,477,398 | 6,676,148,738 | 5,858,349,385 |
| On bait bases | 3,264,345,883 | 3,338,414,084 | 3,404,027,532 | 3,054,289,596 | 3,192,231,463 | 3,481,575,513 | 3,494,483,661 | 3,179,299,216 |
| Near bait bases | 1,654,123,768 | 1,626,706,071 | 1,767,551,356 | 1,506,133,829 | 1,656,127,727 | 1,667,584,908 | 1,838,909,132 | 1,452,697,759 |
| % on/near bait | 80.67 | 80.17 | 79.48 | 79.89 | 80.44 | 80.16 | 79.89 | 79.07 |
| Mean target coverage | 63.21 | 64.71 | 65.81 | 59.32 | 61.56 | 67.57 | 67.54 | 61.92 |
| % target read > 1x | 88.52 | 87.64 | 86.98 | 87.89 | 85.85 | 87.15 | 88.17 | 89.82 |
| % target read > 10x | 63.31 | 61.82 | 61.68 | 64.36 | 61.01 | 61.17 | 61.40 | 63.82 |
| % target read > 20x | 58.91 | 57.85 | 57.76 | 59.68 | 57.12 | 57.43 | 57.54 | 59.20 |
| % target read > 30x | 56.25 | 55.51 | 55.40 | 56.73 | 54.74 | 55.21 | 55.25 | 56.43 |
| % target read > 50x | 52.04 | 51.79 | 51.71 | 51.81 | 50.89 | 51.77 | 51.76 | 52.04 |
