# Supplementary material for: Targeted exome sequencing of unselected heavy‐ion beam‐irradiated populations reveals less‐biased mutation characteristics in the rice genome
Source: Plant J. 2019 Feb 25;98(2):301–14. doi: 10.1111/tpj.14213 (PMC6850588; doi:10.1111/tpj.14213)
Supplement: Supplementary file 8 — Table S5. Survival and mutation frequencies in the M2 generation after irradiation of carbon‐ion beam (LET: 23–30 keV μm−1) to dry seeds. [file TPJ-98-301-s008.pdf]

Table S5. Survival and mutation frequencies in the M_2 generation after irradiation of carbon-ion beam (LET: 23–30 keV/μm) to dry seeds

Dose (Gy)	Survival rate (%)	Infertility rate (%)	Mutation rate (%)
Experiment 1. An approximate tendency of the infertility rate and mutation frequencies in the field (FY2012; n=100)			
0	98.0	0.0	0.0
75	96.0	0.0	4.2
100	98.0	1.0	3.1
125	98.0	5.1	8.7
150	95.0	16.9	11.6
175	98.0	20.1	14.3
200	96.5	24.3	8.8
Experiment 2. Field confirmation of the infertility rate and mutation frequencies in different year (FY2015; n=300)			
0	90.0	0.0	0.0
150	82.5	13.1	10.6
175	79.5	23.2	14.6

In each experiment, the data was an average of two independent experiments in the field. Survival rate, which is the percentage of the M_1 lines grown from the irradiated seeds in a paddy field, was comparable to that of the non-irradiated Nipponbare control up to 200 Gy in the first year (FY2012). In the second year (FY2015), the survival rates were entirely low due to the inadequate weather conditions during the season.
