# Supplementary material for: Targeted exome sequencing of unselected heavy‐ion beam‐irradiated populations reveals less‐biased mutation characteristics in the rice genome
Source: Plant J. 2019 Feb 25;98(2):301–14. doi: 10.1111/tpj.14213 (PMC6850588; doi:10.1111/tpj.14213)
Supplement: Supplementary file 9 — Table S6. Chromosomal distribution of mutations in M2 progenies following 150 Gy irradiation of dry seeds. [file TPJ-98-301-s009.pdf]

Table S6. Chromosomal distribution of mutations in M_2 progenies following 150 Gy irradiation of dry seeds

Chromosome	Chromosome length	Num. of mutations detected	Per Mb chromosome ($\times 10^{-2}$ per line)	Total target length	Per Mb target (per line)
Chromosome 1	43,270,923	127	2.10	10,592,691	0.86
Chromosome 2	35,937,250	127	2.52	8,504,663	1.07
Chromosome 3	36,413,819	109	2.14	9,313,635	0.84
Chromosome 4	35,502,694	74	1.49	6,589,053	0.80
Chromosome 5	29,958,434	96	2.29	5,986,230	1.15
Chromosome 6	31,248,787	76	1.74	5,980,479	0.91
Chromosome 7	29,697,621	62	1.49	5,734,166	0.77
Chromosome 8	28,443,022	63	1.58	4,971,723	0.91
Chromosome 9	23,012,720	52	1.61	4,013,198	0.93
Chromosome 10	23,207,287	64	1.97	3,876,605	1.18
Chromosome 11	29,021,106	78	1.92	4,657,012	1.20
Chromosome 12	27,531,856	69	1.79	4,261,278	1.16
Average ± SD*			1.89 ± 0.33		0.98 ± 0.16

* Standard deviation
