# Supplementary material for: Targeted exome sequencing of unselected heavy‐ion beam‐irradiated populations reveals less‐biased mutation characteristics in the rice genome
Source: Plant J. 2019 Feb 25;98(2):301–14. doi: 10.1111/tpj.14213 (PMC6850588; doi:10.1111/tpj.14213)
Supplement: Supplementary file 10 — Table S7. XHMM output and their visual inspection results. [file TPJ-98-301-s010.pdf]

Table S7. XHMM output and their visual inspection results

SAMPLE	CNV	INTERVAL	KB	CHR	MID_BP	TARGETS	NUM_TAR	Q_EXACT	Q_SOME	Q_NON_DI	Q_START	Q_STOP	MEAN_RD	MEAN_OR	Judgement
13C3-43	DEL	chr05:14409311-14419812	10.5	chr05	14414561	52071..52080	10	21	97	99	21	12	-5.62	33.03	Absent
13C3-50	DUP	chr07:22651231-22696635	45.41	chr07	22673933	69818..69855	38	3	99	99	4	3	3.31	71.68	Absent
13C3-51	DEL	chr08:12797847-12798366	0.52	chr08	12798106	75373..75374	2	16	30	30	14	15	-5.87	185.97	Absent
13C3-55	DEL	chr04:1977055-4547310	2570.26	chr04	3262182	40302..40501	200	1	99	99	11	17	-2.06	37.05	Absent
13C3-55	DEL	chr04:4674698-4876720	202.02	chr04	4775709	40508..40521	14	5	99	95	12	4	-2.61	49.36	Absent
13C3-57	DEL	chr06:10345044-10451010	105.97	chr06	10398027	60373..60404	32	18	98	99	6	6	-2.44	43.06	Absent
13C3-58	DEL	chr04:17492480-18098118	605.64	chr04	17795299	41848..41891	44	6	98	99	28	4	-7.33	20.05	Absent
13C3-58	DEL	chr12:15473909-16198117	724.21	chr12	15836013	98669..98707	39	21	99	99	27	6	-3.27	39.49	Absent
13C3-61	DUP	chr07:11162465-11162783	0.32	chr07	11162624	67513..67514	2	5	31	31	5	30	5.92	167.71	Absent
13C3-62	DEL	chr06:22024348-22139206	114.86	chr06	22081777	62118..62158	41	4	98	99	8	4	-4.31	47.7	Absent
13C3-64	DUP	chr12:20541779-20542019	0.24	chr12	20541899	99257..99257	1	31	31	31	17	31	10	458.76	Absent
13C3-69	DEL	chr07:23620977-23627654	6.68	chr07	23624315	70217..70227	11	43	99	99	9	37	-5.16	30.66	Absent
13C3-71	DUP	chr11:5422223-5422589	0.37	chr11	5422406	91632..91633	2	45	45	45	16	22	6.59	93.2	Absent
13C3-72	DEL	chr10:6404480-7677308	1272.83	chr10	7040894	85535..85686	152	4	99	99	8	5	-2.44	33.72	Absent
13C3-74	DEL	chr09:4105238-4429927	324.69	chr09	4267582	79798..79837	40	25	99	99	29	7	-2.44	41.29	Absent
13C3-74	DUP	chr12:1557212-1558158	0.95	chr12	1557685	96208..96212	5	16	33	33	31	15	3.41	123.83	Absent
13C3-75	DUP	chr08:28293689-28349984	56.3	chr08	28321836	79152..79173	22	6	73	73	7	5	2.12	66.01	Absent
13C3-75	DUP	chr08:28370572-28408839	38.27	chr08	28389705	79180..79210	31	9	99	99	5	9	2.18	59.12	Absent
13C3-78	DEL	chr12:20541779-20542019	0.24	chr12	20541899	99257..99257	1	31	31	31	30	31	-10	401.82	Absent
13C3-81	DEL	chr10:21369911-21570481	200.57	chr10	21470196	88918..89013	96	4	99	99	18	4	-3.82	39.1	Absent
13C3-82	DUP	chr08:12797847-12798366	0.52	chr08	12798106	75373..75374	2	17	40	40	25	12	6.25	255.7	Absent
13C3-82	DUP	chr12:20308195-20542019	233.82	chr12	20425107	99256..99257	2	5	34	34	5	27	6.39	322.34	Absent
13C3-83	DUP	chr08:12797847-12798366	0.52	chr08	12798106	75373..75374	2	22	45	45	19	22	6.44	278.02	Absent
13C3-83	DUP	chr12:20541779-20542019	0.24	chr12	20541899	99257..99257	1	31	31	31	8	31	10	504.15	Absent
13C3-84	DEL	chr01:11813967-12131075	317.11	chr01	11972521	4203..4242	40	13	99	99	17	15	-5	21.19	Absent
13C3-84	DEL	chr04:17180496-18216312	1035.82	chr04	17698404	41835..41910	76	27	98	99	7	33	-4.84	25.3	Absent
13C3-85	DEL	chr01:35505764-35518982	13.22	chr01	35512373	11166..11174	9	30	99	99	6	14	-3.17	49.5	Absent
13C3-88	DUP	chr08:12797847-12798366	0.52	chr08	12798106	75373..75374	2	7	30	30	22	7	5.85	241.4	Absent
13C3-89	DEL	chr07:2940527-2943668	3.14	chr07	2942097	65814..65816	3	24	31	31	27	14	-4.59	78.69	Absent
13C3-97	DEL	chr07:20562504-20705484	142.98	chr07	20633994	69247..69263	17	50	99	99	26	19	-5.65	20.84	Absent
13C3-97	DEL	chr10:15454918-15622656	167.74	chr10	15538787	86956..86965	10	17	99	99	17	21	-3.69	22.11	Absent
13C3-99	DEL	chr09:22927705-22938737	11.03	chr09	22933221	84625..84635	11	5	80	80	27	5	-2.66	84.46	Absent
13C3-101	DUP	chr05:29139220-29141755	2.54	chr05	29140487	56764..56769	6	4	34	34	13	4	3.07	82.61	Absent
13C3-103	DEL	chr01:37891680-37896511	4.83	chr01	37894095	12137..12140	4	7	31	31	7	23	-3.77	41.97	Absent
13C3-103	DEL	chr07:16013069-16031584	18.52	chr07	16022326	68215..68228	14	3	99	99	23	3	-2.65	46.94	Absent
13C3-104	DEL	chr09:16738267-17083380	345.11	chr09	16910823	81973..82087	115	21	99	99	2	10	-2.92	35.47	Absent
13C3-104	DUP	chr12:20308195-20542019	233.82	chr12	20425107	99256..99257	2	29	60	60	23	22	7.45	274.75	Absent
13C3-106	DEL	chr01:4765469-4766402	0.93	chr01	4765935	2051..2053	3	29	58	58	24	10	-5.23	76.33	Absent
13C3-107	DEL	chr01:19302590-19476739	174.15	chr01	19389664	5526..5571	46	17	99	99	4	6	-2.33	53.58	Absent
13C3-114	DEL	chr01:4848955-4854197	5.24	chr01	4851576	2083..2089	7	54	99	99	3	42	-5.6	41.63	Absent
13C3-114	DEL	chr12:23765317-23992564	227.25	chr12	23878940	100033..100141	109	4	99	99	4	15	-3.73	38.83	Absent
13C3-115	DEL	chr12:11716349-11743423	27.07	chr12	11729886	98302..98317	16	5	99	99	5	27	-3.25	41	Absent
13C3-116	DEL	chr07:10498473-10500825	2.35	chr07	10499649	67442..67444	3	48	61	61	24	28	-5.16	24.74	Absent
13C3-116	DEL	chr08:12797847-12798366	0.52	chr08	12798106	75373..75374	2	19	41	41	15	18	-6.29	167.47	Absent
13C3-116	DEL	chr12:20541779-20542019	0.24	chr12	20541899	99257..99257	1	32	32	32	6	29	-10	364.32	Absent
13C3-117	DUP	chr08:752397-758308	5.91	chr08	755352	73012..73016	5	11	54	54	11	19	3.71	106.16	Absent
13C3-117	DEL	chr12:20541779-20542019	0.24	chr12	20541899	99257..99257	1	32	32	32	5	29	-10	363.91	Absent
13C3-118	DUP	chr08:12731867-12798366	66.5	chr08	12765116	75372..75374	3	4	37	37	4	9	4.65	182.71	Absent

13C3-118	DUP	chr12:20308195-20542019	233.82	chr12	20425107	99256..99257	2	23	53	53	12	40	7.2	357.63	Absent
13C3-119	DEL	chr02:32688927-32704634	15.71	chr02	32696780	24948..24959	12	10	99	99	10	4	-5.15	34.87	Absent
13C3-120	DEL	chr01:14294181-14511227	217.05	chr01	14402704	4641..4701	61	6	99	99	4	3	-1.93	51.89	Absent
13C3-120	DEL	chr07:18228994-18252381	23.39	chr07	18240687	68634..68651	18	13	99	99	10	28	-4.59	31.45	Absent
13C3-120	DEL	chr09:22563953-22623187	59.23	chr09	22593570	84376..84404	29	5	99	99	11	4	-6.48	26.14	Absent
