# Supplementary material for: Targeted exome sequencing of unselected heavy‐ion beam‐irradiated populations reveals less‐biased mutation characteristics in the rice genome
Source: Plant J. 2019 Feb 25;98(2):301–14. doi: 10.1111/tpj.14213 (PMC6850588; doi:10.1111/tpj.14213)
Supplement: Supplementary file 12 [file TPJ-98-301-s012.docx]

# Supporting Information

**Figure S1.** Confirmation of a large (102,158 bp) deletion in the 6-62 mutant

**Figure S2.** Determination of survival rate with different irradiation dose

**Figure S3.** Size distribution of deletions and insertions in an unselected carbon-ion beam-irradiated rice population

**Table S1.** Sequencing statistics for individually captured carbon- and neon-ion beam-induced mutants (ordered by LET)

**Table S2.** Verification of the detected variants by PCR and Sanger sequencing

**Table S3.** Covered bases (“Covered”), average read depth (“Average depth”), and the fraction of covered bases against the target region (“Frac.”) in the 6-62 mutant and the average of three mutants (3-14, 7-30, and 7-3B)

**Table S4.** Sequencing statistics for eight mutants for pre-multiplexed target enrichment

**Table S5.** Survival and mutation frequencies in the M_2_ generation after irradiation of carbon-ion beam (LET: 23–30 keV/μm) to dry seeds

**Table S6.** Chromosomal distribution of mutations in M_2_ progenies following 150 Gy irradiation of dry seeds

**Table S7.** XHMM output and their visual inspection results

**Supplemental Data.** List of target exons in IRGSP Build 5 and Os-Nipponbare-Reference-IRGSP-1.0 sequences
